# HIV/AIDS-related attitudes and oral impacts on daily performances: a cross-sectional study of Sudanese adult dental patients

**DOI:** 10.1186/1472-6963-13-335

**Published:** 2013-08-26

**Authors:** Elwalid F Nasir, Mihaela C Marthinussen, Anne N Åstrøm

**Affiliations:** 1 Department of Clinical Dentistry, University of Bergen, Bergen, Norway; 2 Faculty of Dentistry, University of Science and Technology, Omdurman, Sudan

**Keywords:** HIV, AIDS, Attitudes, Oral impacts on daily performance, Dental, Sudan

## Abstract

**Background:**

Few studies have investigated the relationships between HIV-related knowledge, fear of contagion in dental environments and Oral Impacts on Daily Performance (OIDP) among dental patients. Our objectives were to investigate the associations between HIV-related knowledge and fear of contagion in dental environments and OIDP among dental patients, and to evaluate whether those associations were modified by the frequency of dental service attendance.

**Methods:**

A total of 1262 patients (mean age 30.7 years, 56.5% females) were recruited from the Khartoum Dental Teaching Hospital and the University of Science and Technology during March–July 2008. The participants underwent a full-mouth oral clinical examination and completed an interview in a face-to-face setting.

**Results:**

Of the study participants, 41.4% had visited a dentist at least twice during the last 2 years, 96.2% had caries experience (DT > 0) and 79.1% reported oral impacts (OIDP > 0). The most frequently reported oral impacts were problems eating, sleeping and cleaning teeth. In total, 26.3% of the participants had HIV transmission knowledge, 75.6% knew people with HIV/AIDS and 58.7% perceived a high risk of cross-infection in dental environments. After adjusting for sociodemographic characteristics, frequency of dental service attendance and caries experience, patients who had high HIV-related information exposure, a positive attitude toward people with HIV/AIDS and a high perceived risk of cross-infection were more likely to report oral impacts, whereas patients who knew people with HIV/AIDS were less likely to report oral impacts. The association between OIDP and HIV transmission knowledge was modified by frequency of dental service attendance.

**Conclusions:**

Dental patients who were informed about HIV and had a high HIV/AIDS risk perception were more likely to report impaired oral health-related quality of life than their less informed counterparts and those who perceived a low risk of contagion. The effect of HIV transmission knowledge on oral impacts was influenced by frequency of dental service attendance.

## Background

In Sudan, the largest country in sub-Saharan Africa, the prevalence of HIV and AIDS remains low, with a seroprevalence of 1.6% in 2002 [1]. Being bordered by countries with high prevalence of HIV and AIDS, and having experienced long-term ethnic and political conflict, Sudan is highly vulnerable to an increase in the prevalence of HIV and AIDS [1]. Moreover, it is likely that an increase in the prevalence of HIV and AIDS will occur because of decreased mortality rates and increased incidence. Since dental treatment is an important part of HIV/AIDS disease management, patients are expected to present at Sudanese dental health care services with an increasing frequency. The ability of the Sudanese dental health system to cope with this expected increase in demand for services by the HIV-infected persons remains questionable. A previous study revealed that Sudanese dental students receive a limited amount of HIV/AIDS-related information, and that they were not appropriately prepared for treating patients with HIV/AIDS [2]. Fear of cross-infection among dental health care personnel, attributed to a lack of proper knowledge about HIV and its transmission route, might lead to an unwillingness to treat patients with HIV/AIDS altogether [3]. In a study from Mexico, about one-third of the dentists investigated did not intend to treat patients with HIV-infection [4]. Fear of losing non-HIV positive patients was one of the main reasons given for refusing treatment to patients with HIV [4]. Studies from the US and Canada have revealed that dentists in those countries have the same fear [3,5]. In part, the problem might be related to the public’s attitudes and fear of HIV contagion in dental environments [6-10].

A previous survey focusing on dental patients in Khartoum showed that only a minority supported treatment of patients with HIV infection in general dental clinics [11]. Humphris *et al.*[9] reported that one-third of regular dental service attendees in the United Kingdom believed that there was at least a slight risk of contracting HIV at dental clinics. Studies from different parts of the world have reported variable results in terms of the percentages of respondents having fear of cross infection for HIV and AIDS in dental practice and negative attitudes towards HIV infected patients [12-15]. In a Nigerian study of public perceptions of cross-infection control in dentistry, more than half of the respondents felt that they could contract an infection in the dental clinic and 43% identified HIV as a risk [10]. Pistorius *et al.*[7] examined dental patients in Germany and found that about 17% were generally afraid of contracting an infection in the dental environment. Thomson *et al.*[8] examined perceptions of cross-infection in dentistry among Australians and found that 3.6% reported delayed or avoided dental visits because of the perceived risk of cross-infection. In that study, the avoidance rate was highest among those who reported concerns about cross-infection control. A Mexican study revealed that only 21.2% of study participants intended to continue treatment at a dental practice where patients with HIV were treated or where the dentist was HIV-positive [6]. Evidence suggests that Sudanese dental patients who are well informed about HIV transmission routes and who have experience with people with HIV/AIDS tend to seek dental care less frequently than their counterparts after adjusting for differences in oral health status [16]. Numerous studies have demonstrated strong associations between use of dental care services, dentition status and oral health-related quality of life (OHRQoL) [17,18]. However, the implications of patients’ HIV-related knowledge and fear of contagion on their OHRQoL, and the role of dental service attendance as a possible modifier of that relationship, remain unknown. OHRQoL is a multidimensional construct referring to the extent to which oral diseases impact on individuals’ normal functioning and daily performances [19]. This concept is increasingly recognized to be an essential component of oral health surveys, clinical trials and other studies evaluating the outcomes of therapeutic and preventive programs implemented to improve oral health [20]. Impacts on daily performances might be assessed by the Oral Impacts on Daily Performances (OIDP), designed to measure the impacts that seriously affect a person’s daily life. Since its development [21], the OIDP has been used in epidemiological studies of adult populations and numerous studies have examined OIDP in relation to clinical and subjective oral health indicators [22].

### Purpose

Focusing on adult dental patients with unknown HIV status in Khartoum State, this study investigated the relationship between HIV-related knowledge, attitudes and fear of contagion in the dental environments and OHRQoL, and whether that association was modified by the use of dental services.

## Methods

### Study participants

The cross-sectional study presented was carried out from March to July 2008. Survey participants were recruited from dental clinics at two teaching hospitals in Khartoum, Khartoum Dental Teaching Hospital (KDTH) and University of Science and Technology (UST). In both hospitals, all patients coming with dental complains are registered and then examined at the outpatients ‘diagnostic’ department for oral examination. All patients between 20 and 60 years of age with reported unknown HIV status were invited to participate in the study. Exclusion criteria were; patients presented with severe pain and/or emergency were excluded. A total of 769 patients in KDTH (response rate 769/2650, 29.0%) and 491 patients in UST (response rate 491/950, 52%) consented to participate in a clinical examination, saliva sampling for a HIV test and an interview. Pre-test and post-test counselling was arranged before the conduction of the study. Reason for not participating was mainly due to time constraints on the part of patients and eagerness to receive the dental treatment. A sample size of 1200 patients was assumed to be satisfactory for a two-sided test assuming the proportion of dental care utilization in the previous 2 years to be 0.15 and 0.20 in patients with respectively low- and high education, a significance level of 5% and a power of 95%. Ethical approval was obtained from the Norwegian Regional Ethical Committee, Sudan National AIDS Programme (SNAP) and from the UST, and KDTH prior to conduction of the study.

### Oral examination

One trained and calibrated dentist (EFN) conducted all clinical examinations in a dental clinic equipped with an adjustable dental chair and artificial lightning. Examinations were conducted using disposable gloves, sterilized dental mirrors, periodontal probes and dental explorers. Dental caries were recorded using the Decayed, Missing, Filled Teeth (DMFT) index, according to the WHO guidelines [23] and re-corded as 0 or 1 (DMFT = 0 no caries experience, DMFT > 0 caries experience). Duplicate clinical examinations for dental caries, 2 months apart, were carried out among 14 chair-side dental assistants at UST. Intra-examiner reliability in terms of Cohen’s kappa for the DMFT component was 1 (100%).

### Interviews

In addition to the full mouth oral clinical examination, structured face-to-face interviews were conducted with respect to sociodemographic characteristics, HIV transmission knowledge, HIV-related information exposure, HIV/AIDS-related attitudes, and previous experience with HIV/AIDS, perceived risk of HIV/AIDS and perceived severity of HIV. The interview was constructed in English, translated into Arabic by a dentist, and then re-translated back to English by another dentist to check for consistency in the language. Two dentists (a male and a female) were assigned and trained to carry out the interviews. Patients were interviewed in a confidential atmosphere while waiting for their clinical examination.

### Independent variables

Sociodemographic data (age, gender, and hospital attended) were collected (Table 1). HIV/AIDS-related attitudes and fear of contagion in dental environments were assessed in terms of: 1) amount of HIV information received from various sources; 2) knowledge of HIV transmission routes; 3) previous experience with people with HIV/AIDS; 4) attitudes toward people with HIV/AIDS; 5) perceived personal risk of contracting HIV/AIDS as a dental patient; and 6) perception of HIV as a dangerous disease. *Amount of HIV/AIDS information received* was assessed by four questions: “How much information about HIV have you received from: 1) radio/TV; 2) reading materials; 3) friends/relatives; and 4) health care workers.” Each question had response categories ranging from (1) “little” to (5) “very much”. For cross-tabulation each question was dichotomized into (0) some/a little received (original categories 1, 2, 3) and (1) much/very much information received (original categories 4, 5). A formative summary score was constructed and dichotomized based on a median split yielding (1) very much/much HIV information received and (0) some/little/no HIV information received. *Knowledge about modes of HIV transmission* was assessed using the statements: “HIV can be transmitted by: 1) using contaminated sharp instruments; 2) unsafe blood transfusion; 3) shaking hands; 4) eating with infected people.” Each statement had response categories ranging from (1) “strongly agree” to (5) “strongly disagree.” Each statement was dichotomized yielding (1) correct knowledge (original categories 4, 5) and (0) incorrect knowledge (original categories 1, 2, 3). A summary transmission knowledge score was constructed from the four dummy variables and dichotomized based on a median split into (1) correct overall knowledge and (0) incorrect overall knowledge. *Previous experience with HIV/AIDS* was assessed using three items: 1) “Have you personally known anyone who is HIV-positive?” 2) “Have you personally known anyone who is sick with AIDS?” 3) “Have you known anyone who has died because of AIDS?” Response categories were (1) “yes” and (0) “no.” A summary previous experience score was constructed from the three dummy variables and dichotomized based on a median split into (0) for no experience and (1) for experience. *Attitudes toward people with HIV/AIDS* were assessed by four statements: 1) “I would go and visit a friend/relative if I knew that he/she had HIV/AIDS”; 2) “I would continue to be friends with someone who got infected with HIV”; 3) “If a member of my family became sick with HIV/AIDS I would want this to remain a secret”; 4) “I would be willing to take care of someone with HIV/AIDS.” Responses were given on five-point Likert scales from (1) “strongly disagree” to (5) “strongly agree.” Dummy variables for (0) negative and (1) positive attitudes were constructed and summed. The summary score was dichotomized based on a median split into (0) for negative attitude toward people with HIV/AIDS and (1) for positive attitude toward people with HIV/AIDS. *Perceived personal risk of contracting HIV/AIDS* was assessed by one question, “How do you rate your own risk as a dental patient of contracting HIV/AIDS when attending a dental practice?” Responses were given on a scale ranging from (1) “no risk” to (4) “great risk” and dichotomized into (0) low risk (original categories 1, 2) and (1) high risk (original categories 3, 4). *Perception of HIV as a dangerous disease* was assessed by one statement, “HIV is the most dangerous disease in Sudan.” Responses were given on a five-point Likert scale from (1) “strongly disagree” to (5) “strongly agree.” Dummy variables, (0) no and (1) yes, were constructed and summed. The summary score was dichotomized based on a median split into (0) no and (1) yes. *Teeth condition* was assessed by one question, “How do you consider the present condition of your mouth and teeth?” with the response categories, (1) “good” and (0) “bad.” *Frequency of dental service attendance* was assessed by asking, “During the past two years, how often have you attended a dental clinic in order to receive treatment?” Responses were given as (0) “once” or (1) “twice or more.”

**Table 1:** Frequency distribution of independent variables (n = 1262)

**Characteristic**	**% (n)**
**Hospital of attendance**	
UST	39.0 (491)
KDTH	61.0 (769)
**Gender**	
Male	43.5 (548)
Female	56.5 (712)
**Age group**	
≤29 year	54.2 (682)
≥30 year	45.8 (577)
**Dental attendance last 2 years**	
Once	58.6 (693)
Twice and more	41.4 (490)
**Caries experience**	
DT = 0	3.8 (45)
DT > 0	96.2 (1146)
**HIV information received**	
Little	60.6 (762)
A lot	39.4 (496)
**Knowledge on HIV transmission routes**	
Little	73.7 (928)
High	26.3 (332)
**HIV experience**	
Yes	24.4 (308)
No	75.6 (952)
**Attitudes HIV infected patients**	
Negative	50.9 (641)
Positive	49.1 (618)
**Perceived risk of HIV transmission**	
Low	41.3 (521)
High	58.7 (739)
**HIV dangerous disease**	
Yes	11.5 (144)
No	88.5 (1113)
**OIDP**	
= 0	20.9 (262)
>0	79.1 (990)

### Dependent variable

OHRQoL was assessed using the eight-item Oral Impacts on Daily Performance (OIDP) frequency inventory [21,24]. To be administered among adult dental patients in Khartoum, a translation into the Arabic language was necessary. Project staff reviewed the Arabic version of the OIDP for semantic, experimental and conceptual equivalence with the source version. Sensitivity to culture and selection of appropriate words were considered. One OIDP item (problems speaking and pronouncing clearly) was removed from the scales because of difficulties with the translation process. The following seven OIDP items were used in the interview: “During the past six months, how often have problems with your mouth and teeth caused you any difficulty with *eating and chewing food*, *cleaning teeth*, *sleeping and relaxing*, *smiling and showing teeth without embarrassment*, *maintaining usual emotional state*, *carrying out major work and social roles*, and *enjoying contact with people*?” Each item was assessed using a five-point scale: (1) “never affected”; (2) “less than once a month”; (3) “once or twice a month”; (4) “once or twice a week” and (5) “every or nearly every day”. A summary score (OIDP ADD) was constructed from the seven items as originally scored (0–5) (range 7–35). Each OIDP frequency item was dichotomized, yielding the categories (0) never affected (including the original category 1) and (1) affected (including the original categories 2, 3, 4, and 5). Simple count scores (SC) were created for the OIDP by adding the seven dichotomized variables. For the purposes of cross-tabulation and logistic regression analysis, and to assess the extent of OIDP (), the OIDP SC scores (0–7) were dichotomized as (0) no daily performance affected and (1) at least one daily performance affected. The distribution of the OIDP SC scores supported this cut-off point. This is in accordance with a standard method to handle the OIDP score [25,26].

### Statistical methods

Data were analyzed using the Statistical Package for Social Sciences version 15.0 (SPSS Inc., Chicago, Illinois, USA). Bivariate analyses were conducted using cross-tabulations, Chi-squared statistics and Mann Whitney *U* tests. Internal consistency reliability was assessed using Cronbach’s alpha. The associations between HIV-related attitudes and OIDP were initially examined by multiple logistic regression analyses accounting for the potential confounding effects of socio-demographic characteristics, tooth decay and dental service attendance. In the final logistic regression model, all HIV-related attitude indicators were adjusted for in addition to socio demographics, tooth decay and dental service attendance.

## Results

A total of 1262 dental patients participated in the study. Table 1 presents their socio-demographic characteristics, HIV-related information exposure, attitudes and experience, dental service attendance, caries experience and OIDP scores. Participants’ mean age was 30.7 years (SD 8.5), 56.5% were females and 61.0% were from KDTH. Of the participants, 42.9% were residents of Omdurman City, 31.4% of Khartoum City, 16.2% of Khartoum North City and 9.5% of other states. In total, 41.4% had visited a dentist twice or more during the last 2 years; 96.2% had caries experience (DT > 0); 26.3% had HIV transmission knowledge; 75.6% had no previous experience with people with HIV/AIDS; 50.9% reported negative attitudes toward people with HIV/AIDS; 58.7% perceived a high risk of being infected with HIV/AIDS at a dental practice and 88.5% did not consider HIV to be a dangerous disease.

Oral impacts (OIDP > 0) were reported by 79.1% (mean 2.9 sd = 1.3) (85.3% in UST and 75.2% in KDTH) of the participants. The most frequently reported oral impacts were eating (67.1%), sleeping (56.6%) and cleaning (41.6%) as shown in Table 2. Internal consistency reliability (standardized item alpha) in terms of Cronbach’s alpha was 0.82. As shown in Table 3, construct and discriminative validity of the OIDP was confirmed in that the OIDP statistically significantly discriminated between groups with and without decayed teeth (80.2% versus 69.5%, p < 0.01), missing teeth (81.0% versus 75.8%, p < 0.05) and filled teeth (73.5% versus 80.0%, p < 0.05). It also discriminated between groups with and without frequent dental service attendance patterns and good and bad teeth condition (p < 0.01). ANOVAs with mean OIDP group differences revealed the same results.

**Table 2:** Frequency distribution of OIDP items and summed OIDP scores by hospital attended

**OIDP items**	**UST**	**KDTH**	**TOTAL**
**Eating & chewing**	71.6% (351)*	64.1% (492)	67.1% (843)
**Cleaning**	48.3% (237)**	37.3% (286)	41.6% (523)
**Sleeping**	61.1% (300)**	53.7% (412)	56.6% (712)
**Laughing**	31.4% (154)	27.1% (208)	28.8% (362)
**Emotional state**	30.9% (151)	27.6% (212)	28.9% (363)
**Social role**	36.7% (179)*	29.7% (228)	32.4% (407)
**Social contact**	37.0% (180)*	31.2% (239)	33.4% (419)
**Sum OIDP**	85.3% (413)**	75.2% (577)	79.1% (990)

*p < 0.05, **p < 0.01.

Percentages (n) of those reporting oral impacts.

**Table 3:** Percentages (n) and mean (sd) OIDP by tooth condition, decayed teeth, missing teeth, filled teeth and dental attendance

	**% (n)**	**Mean (sd)**
**Tooth condition**		
Good	70.3 (496)	2.2 (2.1)
Bad	90.5 (494)**	3.6 (2.2)**
**Decayed teeth**		
None	69.5 (89)	2.3 (2.3)
At least one	80.2 (901)**	2.9 (2.2)
**Missing teeth**		
Non	75.8 (357)	2.5 (2.1)
At least one	81.0 (633)*	3.1 (2.3)*
**Filled teeth**		
None	80.0 (857)	2.9 (2.3)
At least one	73.5 (133)*	2.6 (2.2)
**Dental attendance last 2 years**		
Once	72.3 (501)	2.4 (2.2)
Twice or more	89.0 (435)**	3.5 (2.3)**

*p < 0.05, **p < 0.01.

Note: The OIDP statistically significantly discriminated between groups with and without decayed teeth, indicating discriminative and construct validity.

Table 4 presents summary of logistic regression models whereby OIDP was regressed on HIV information exposure, HIV transmission knowledge, HIV/AIDS experience, attitudes toward people with HIV/AIDS, perceived risk of HIV/AIDS and perception of HIV as a dangerous disease. Each regression model considering OIDP was adjusted for socio-demographic characteristics, dental service attendance and tooth decay. As shown in Table 4, more HIV information exposure (OR 1.5, 95% CI 1.1–2.1), positive attitude toward people with HIV/AIDS (OR 1.4, 95% CI 1.1–1.9) and high perceived risk of HIV/AIDS (OR 1.2, 95% CI 1.0–1.7) increased the odds of having an oral impact (OIDP > 0). Having experience with HIV/AIDS decreased the odds of having an oral impact (OR 0.5, 95% CI 0.3–0.8). A multivariate logistic regression model was fitted with socio demographics entered in the first step, providing a R^2^ of 0.08. Entering HIV information exposure, HIV/AIDS experience, attitude toward people with HIV/AIDS and perceived risk of HIV/AIDS in a second step raised the explained variance to 11% (R^2^ 0.11). Entering dental caries (using the deuced component DT) and dental attendance in a third step increased the explained variance to 16% (R^2^ 0.16). Most variables remained statistically significantly associated with OIDP in the final model. The OR of having any oral impact was 2.2 (95% CI 1.6–3.1) for females; 1.4 (95% CI 1.0–2.0) for high HIV information exposure; 1.3 (95% CI 1.0–1.8) for having a positive attitude toward people with HIV/AIDS; 2.1 (95% CI 1.0–4.3) for having dental caries experience and 2.5 (95% CI 1.8–3.6) for being a frequent dental service attendee. The perceived risk of HIV/AIDS infection did not remain statistically significantly associated with OIDP after DT and dental service attendance were added to the model (Table 5). Two-way interactions between frequency of dental service attendance and HIV information on OIDP and between frequency of dental service attendance and HIV transmission knowledge on OIDP were statistically significant (p < 0.05). Table 6 presents adjusted logistic regression analyses stratified by frequency of dental service attendance. Among less frequent dental service attendees, but not among frequent dental service attendees, receiving a lot of HIV information increased the likelihood of reporting any OIDP. In contrast, among frequent dental service attendees, but not among less frequent attendees, having HIV transmission knowledge increased the likelihood of reporting any oral impact.

**Table 4:** Summary of logistic regression analyses of factors associated with OIDP

**Each independent adjusted for SES and DT**	**Unadjusted% (n)**	**Adjusted OR (95% CI)**^**a**^
Information on HIV		
Little	76.8 (579)	1
A lot	82.5 (409)*	1.5 (1.1-2.1)^a^
**Knowledge on HIV transmission routes**		
Low	79.2 (729)	1
High	79.0 (261)	1.1 (0.7-1.5)^a^
**HIV experience**		
No	87.9 (268)	1
Yes	76.2 (722)**	0.5 (0.3-0.8)^a^
**Attitudes HIV infected patients**		
Negative	76.0 (465)	1
Positive	82.0 (524)*	1.4 (1.1-1.9)^a^
**Perceived risk as**		
Low	76.1 (461)	1
High	81.8 (5219*	1.2 (1.0-1.7)^a^
**HIV dangerous disease**		
Yes	77.8 (112)	1
No	79.4 (877)	1.2 (0.7-1.9)^a^

^a=^Adjusted for age, sex, hospital of attendance, decayed teeth and frequency of dental attendance. *p < 0.05, **p < 0.01.

**Table 5:** OIDP regressed on sociodemographic, attitudes, fear, tooth decay and use of dental services

	**Step I **^**a**^	**Step II **^**b**^	**Step III **^**c**^
**Hospital**			
UST	1	1	1
KDTH	0.5 (0.3-0.7)	0.5 (0.3-0.7)	0.6 (0.4-0.8)
**Sex**			
Male	1	1	1
Female	2.6 (81.9-3.5)	2.5 (1.8-3.4)	2.2 (1.6-3.1)
**Age**			
≤29 years	1	1	1
>30 years	0.7 (0.5-1.1)	0.8 (0.6-1.2)	0.7 (0.5-1.0)
**Information on HIV**			
Little		1	1
A lot		1.4 (1.0-1.9)	1.4 (1.0-2.0)
**HIV experience**			
No		1	1
Yes		0.5 (0.3-0.8)	0.6 (0.4-0.9)
**HIV attitudes**			
Negative		1	1
Positive		1.3 (1.0-1.9)	1.3 (1.0-1.8)
**Perceived risk**			
Low		1	1
High		1.2 (1.0-1.7)^a^	1.2 (0.8-1.6)
**Decayed teeth**			
None			1
At least one			2.1 (1.0-4.3)
**Dental attendance**			
Once			1
At least twice			2.5 (1.8-3.6)

^a=^ Nagelkerke’s R^2^ 0.08, ^b^ Nagelkerke’s R^2^ 0.11, ^c^ Nagelkerke’s R^2^ 0.16.

**Table 6:** Regression of fear of contagion indicators on OIDP, stratified by frequency of dental service attendance

**Indicator**	**Once last 2 years**	**Twice or more last 2 years**
**Information in HIV**		
Little	1	1
A lot	1.9 (1.3-2.7)	1.0 (0.5-1.8)
**Knowledge on HIV transmission routes**		
Low	1	1
High	0.8 (0.5-1.1)	2.9 (1.1-7.6)

## Discussion

This study is the first to assess disparities in the OHRQoL of Sudanese adult dental patients according to their exposure to HIV-related information and perceived risk of HIV/AIDS. The general association between fear of dental care or dental anxiety and OHRQoL has not been frequently examined [27-31]. McGrath and Bedi revealed that people in Britain with dental anxiety were twice as likely as others to be among the group with the poorest OHRQoL [29]. Even fewer studies have considered the influence of fear of HIV/AIDS infection on OHRQoL among people living in non-occidental cultural contexts.

More than half of the dental patients investigated were concerned about the risk of contracting HIV/AIDS in a dental environment, whereas 26% had received very much HIV-related information and 39% were knowledgeable about HIV transmission routes (Table 1). Previous studies from Hong Kong, the United Kingdom and Mexico City have revealed similar rates with respect to patients being concerned about HIV contagion [6,32]. In contrast, studies from the United Kingdom have shown that participants believe the risk of HIV transmission in dental practices to be very low [9]. This difference might be attributed to the accuracy of information provided in the context of contagion risk and poor compliance with infection control procedures in the clinical settings [33]. A direct comparison between the present and previous findings is difficult because of the use of different instruments with different scopes of focus for measuring risk and concern of HIV infection in dental practices. In general, the prevalence of oral impacts was higher in our study than those reported in various age groups of the general adult population in sub-Saharan countries using the OIDP instrument [26]. However the prevalence among dental patients investigated in this study is similar to those found among adults from the general population presenting with caries experience and mobile teeth [26].

According to the present results, concepts that might reflect a fear of HIV cross-infection at a dental practice, such as exposure to a lot of information about HIV/AIDS from the media (where the risk of getting infection is commonly overstated), and having a high HIV/AIDS risk perception increased the likelihood of reporting any OIDP. The identified associations were of moderate strength even after potential confounders were taken into consideration. Although not conclusive, there is evidence that dental anxiety can lead to avoidance behaviors, resulting in a lack of regular dental care and delay in necessary treatments [12,34]. Other studies have contradicted the suggestion that the dentition of dentally anxious people remains unrestored [35]. Based on cross-sectional data, such relationships might be bidirectional, for example problem-motivated dental visits might be more stressful, with unpleasant treatment experiences, thus reinforcing anxiety and in turn avoidance behavior [12]. It is also widely assumed that the avoidance of dental care might have a detrimental effect on peoples’ OHRQoL [35,36].

As shown in Table 5, the significant association between HIV/AIDS-related risk perception and OIDP disappeared in the final step of the regression, indicating that the effect was mediated through dental caries status and dental service attendance patterns. It is interesting that most fear of infection indicators tended to increase oral impacts, independent of variations in oral health and dental attendance patterns. This is in accordance with evidence suggesting that perceived oral health status is a complex function of sociodemographic and personal factors and that clinically recorded dentition status might not be the primary variable contributing to OHRQoL. Accordingly, previous studies have shown that the reduction of dental fear improves OHRQoL rather than enhances oral health status [37,38]. When mutually adjusted, exposure to HIV-related information, attitudes toward people with HIV/AIDS and perceived risk of HIV/AIDS infection independently contributed to the explained variance in OIDP beyond the contributions of sociodemographic characteristics and oral health indicators. Nevertheless, the model explained only 16% of the explainable variance in OIDP, indicating the importance of other factors not accounted for in the present study, such as culture and aspects of the Sudanese dental health care system itself. A caveat of this study that might have influenced the relationships estimated is the recoding into dummy variables of DMFT and OIDP, originally measured as count variables. This might have led to loss of information and variation in those variables. The significant effect modification of dental service attendance indicated that the relationship between HIV transmission knowledge and oral impacts differed by the frequency of dental service attendance. Being aware of HIV transmission routes increased the likelihood of any OIDP impact among frequent dental service attendees, but not among infrequent dental service attendees. In contrast, Pohjola *et al.*[28] did not confirm any effect modification of the number of remaining teeth on the relationship between dental fear and subjective oral health in Finnish adults. Effect modification was expected because knowledge about HIV/AIDS and HIV transmission routes has been found to act as a barrier towards frequent use of dental services in previous studies using the same sample [16]. A possible explanation for our finding is that different dental care experiences and different types of dental treatment are received among frequent dental service attendees compared with infrequent attendees. In accordance with previous findings from resource-poor settings, frequent dental service attendance increased the likelihood of oral impacts. Thus, because of the limited accessibility of dental health care services, people with significant dental problems and poor OHRQoL seek dental care for symptomatic reasons and receive more unpleasant treatment procedures, which in turn might reinforce their fear of HIV contagion. This agrees with the proposition that OHRQoL is an indicator of service need and intervention outcomes in contemporary public health research and practice [27]. Alternatively, frequent dental service attendees may have been exposed to inaccurate infection control procedures and inaccurate information about transmission risk more extensively than their infrequent dental service attendee counterparts, thus strengthening their fear of contagion and oral impacts.

It is not possible to argue that the present results demonstrate the crude impacts of the various concepts considered, because each association might be biased by residual confounding factors. Because the study participants were patients attending referral hospitals for treatment, individuals who attended care for prophylactic reasons were excluded from the study group. Thus, the recruited participants cannot be considered to be representative of its study population. This might have led to an overestimation of OHRQoL, because excluded dental attendees might have a low need for dental care. They might also possess HIV-related attitudes and perceptions that differ from the participants of the present study. Moreover, because of our recruitment method, those with extreme anxiety have been excluded from the study group because they do not attend subsequent appointments. Thus, it is unclear how close the present estimates are to the situation in the general adult population of Sudan. It should also be noted that to go beyond correlations and establish causal relationships between the fear of contagion and OHRQoL, prospective studies are needed.

## Conclusions

This cross-sectional study revealed that dental patients who were informed about HIV and were concerned about the risk of contagion were more likely to report impaired OHRQoL than their less informed counterparts and those who perceived the risk of contagion to be low. The effect of HIV transmission knowledge on oral impacts was influenced by frequency of dental service attendance. The public need information about the actual risk of HIV cross-infection in dental offices where official infection control regimes have been implemented. Dental health workers should be educated to pass on accurate information about the risk of HIV contagion in dental practices [39]. Such efforts could improve OHRQoL in resource-poor settings bearing the greatest burden of HIV/AIDS in the world.

## Competing interests

The authors declare that they have no competing interests.

## Authors’ contributions

EFN conceived of and designed the study, collected data, performed statistical analysis and drafted the manuscript. MCM was involved in outlining the manuscript and contributed to all writing phases from data analysis. ANÅ was the main supervisor of the present study, participated in and guided the study design and was actively involved in all stages throughout the work, especially the statistical analyses of data. All authors read and approved the final manuscript.

## Pre-publication history

The pre-publication history for this paper can be accessed here:

http://www.biomedcentral.com/1472-6963/13/335/prepub
